# Structure function relationships differ between optic neuritis and glaucoma with comparable optical coherence tomography findings

**DOI:** 10.1371/journal.pone.0353553

**Published:** 2026-07-16

**Authors:** Heesuk Kim, Mi Jeong Kim, Jihei Sara Lee, Chan Yun Kim, Hyoung Won Bae

**Affiliations:** 1 Department of Ophthalmology, Severance Hospital, Institute of Vision Research, Yonsei University College of Medicine, Seoul, Republic of Korea; 2 Department of Ophthalmology, Saevit Eye Hospital, Goyang, Republic of Korea; Brigham and Women's Hospital, UNITED STATES OF AMERICA

## Abstract

This retrospective study compared structure-function relationships between patients with optic neuritis (ON) and primary open-angle glaucoma (POAG), focusing on the extent of retinal nerve fiber layer (RNFL) and ganglion cell-inner plexiform layer (GCIPL) damage and its correlation with visual field (VF) defects. We included 194 patients (ON: 47; POAG: 147) referred to Yonsei University Severance Eye Hospital between 2017 and 2023. RNFL and GCIPL thickness, VF indices, and the relationship between structural and functional measures were assessed. Despite comparable RNFL and GCIPL thinning, ON demonstrated significantly better VF performance than POAG (mean deviation: −2.26 dB vs. −7.32 dB; VF index: 95.48% vs. 80.43%; both p < 0.001). In POAG, VF loss was strongly correlated with structural parameters, whereas in ON, VF remained preserved even at low RNFL and GCIPL values. Linear regression with robust error estimation confirmed significant interaction between disease type and structure–function slopes (p < 0.01). These findings persisted after 1:2 propensity score matching for age and comorbidities (ON: n = 29; POAG: n = 58; all interaction p < 0.05), age-adjusted multivariable regression, and a sensitivity analysis restricted to non-diabetic participants (ON: n = 45; POAG: n = 124; all interaction p < 0.001). This dissociation was evident at RNFL <90 µm and GCIPL <80 µm, where ON showed better VF indices than POAG with similar structural loss. The differences in structure–function relationships underscore the importance of disease-specific diagnostic approaches and unraveling the distinct mechanisms underlying ON and POAG to improve the management of visual impairments.

## Introduction

Optic neuritis (ON) and primary open-angle glaucoma (POAG) are two major causes of optic neuropathy with distinct pathophysiologic mechanisms. ON involves optic nerve inflammation, leading to visual dysfunctions ranging from mild blurring to complete vision loss [1–3]. It is frequently associated with conditions such as multiple sclerosis or neuromyelitis optica spectrum disorder. In contrast, POAG is a chronic neurodegenerative disorder characterized by progressive retinal ganglion cell (RGC) loss [4].

Both diseases involve thinning of the retinal nerve fiber layer (RNFL) and ganglion cell-inner plexiform layer (GCIPL), measurable with optical coherence tomography (OCT). However, their clinical implications differ substantially. In POAG, structural thinning closely aligns with visual field (VF) decline, making OCT a reliable tool for disease monitoring [5,6]. In ON, structural damage may persist despite marked visual recovery, indicating a disconnect between anatomy and function [1,7]. Furthermore, OCT-based structural measurements are subject to a “floor effect,” whereby further neuronal loss may not be detectable once RNFL or GCIPL thickness reaches its lower measurement limit, which complicates cross-disease comparisons at equivalent OCT thickness levels.

Despite the growing use of OCT across optic neuropathies [8,9], few studies have directly compared structure-function relationships between ON and POAG under unified protocols [10]. Such comparison is essential for determining whether disease-specific thresholds are needed for accurate diagnosis and management.

We aimed to analyze and compare the structure–function relationships in ON and POAG by evaluating how RNFL thickness (RNFLT) and GCIPL thickness (GCIPLT) correlate with VF indices. We further assessed whether these relationships exhibit disease-specific patterns, particularly at varying levels of structural damage, using standardized OCT and perimetric protocols. To rigorously account for demographic differences between groups, we additionally performed propensity score (PS)–matched analyses and a pre-specified sensitivity analysis restricted to non-diabetic participants.

## Methods

### Study design and participants

This retrospective, comparative study included 194 eyes of 194 patients who were evaluated at Severance Eye Hospital, Yonsei University College of Medicine, between March 2017 and October 2023. The study protocol adhered to the tenets of the Declaration of Helsinki and received approval from the Institutional Review Board (IRB) of Severance Hospital, Yonsei University College of Medicine (IRB file number: 4-2024-0755). The need for written informed consent was waived by the IRB of Severance Hospital, Yonsei University College of Medicine because of the retrospective study design and use of de-identified patient data. The de-identified clinical data used in this study were accessed for research purposes between 08/08/2024 and 31/12/2024. This study was conducted and reported in accordance with the Strengthening the Reporting of Observational Studies in Epidemiology (STROBE) guidelines. The STROBE checklist was completed to ensure comprehensive reporting of all relevant study components.

### Patient selection

Patients with ON aged 18–67 years followed up for at least 6 months were eligible for inclusion (S1 Fig.; STROBE flowchart). ON was diagnosed if one of the following criteria was met. The first criterion was the patients exhibited acute loss of visual acuity (VA) or VF, accompanied by ocular pain on eye movement, relative afferent pupillary defect, and abnormal color vision on the Ishihara color sense test. Additionally, the patients had abnormal optic disc swelling (identified through fundus examination or OCT) or underwent gadolinium enhancement of the optic nerve on magnetic resonance imaging (MRI). The second criterion was the patients experienced acute loss of VA or VF with relative afferent pupillary defect and abnormal color vision but without ocular pain on eye movement. These cases required the presence of both abnormal optic disc swelling and gadolinium enhancement of the optic nerve on MRI. All ON patients underwent serum analysis for anti-aquaporin-4 (AQP4) and anti-myelin oligodendrocyte glycoprotein (MOG) antibodies using cell-based indirect immunofluorescence assays. Antinuclear antibody (ANA) status was evaluated by indirect immunofluorescence on HEp-2 cells. Patients had visual symptoms lasting <14 days and were treated according to the Optic Neuritis Treatment Trial protocol [11,12].

ON eyes were included only after clinical stabilization, defined as (1) at least 6 months after symptom onset, (2) no change in treatment, (3) no structural (RNFLT/GCIPLT) or functional (mean deviation [MD]/VF index [VFI]) worsening on repeated testing across at least two consecutive visits on the same device beyond expected test–retest variability, and (4) absence of optic disc edema on OCT examination. This comprehensive approach ensures that analyzed measurements represent true post-inflammatory structural changes rather than residual edema or ongoing inflammation.

POAG was defined by characteristic optic disc changes and corresponding glaucomatous VF defects. VF defects were considered significant if any of the following criteria were met: (1) a cluster ≥3 points with p < 5%, with at least one < 1%; (2) pattern standard deviation (PSD) value < 5%; and (3) glaucoma hemifield test outside normal limits. The patients were required to have open angles on gonioscopy. Additionally, the glaucoma had to be controlled with medication (intraocular pressure [IOP] <21 mmHg or at a level deemed safe by the ophthalmologist to prevent further optic nerve damage). Patients with mild, moderate, and severe POAG were included, provided that they fulfilled the predefined diagnostic and stability criteria. For this cross-sectional analysis, we deliberately selected only clinically stable POAG patients to enable fair comparison with post-inflammatory stable ON patients. The last clinically stable visit within the preceding 12 months, requiring no therapy escalation and no evidence of deterioration on repeated OCT/VF testing across ≥ 2 consecutive visits on the same device (i.e., no chart-documented progression and no change beyond expected test–retest variability). Humphrey VF Guided Progression Analysis (GPA) and device-specific OCT progression tools (e.g., Cirrus OCT GPA for RNFLT and GCIPLT) were used in conjunction with clinical medical records to confirm the absence of progression. If both eyes were eligible, one eye was randomly selected for inclusion.

### Ophthalmological evaluation

All patients underwent comprehensive ophthalmic examinations, including measurement of best-corrected VA (BCVA), refraction, axial length, IOP (measured via Goldmann applanation tonometry), and slit-lamp biomicroscopy with dilated fundus examination.

VF was tested using the Humphrey Field Analyzer II (Carl Zeiss Meditec, Inc., Dublin, California, USA) with the Swedish Interactive Threshold Algorithm Standard 24−2 strategy. Only reliable VF results—defined as fixation loss <20% and false-positive and false-negative rates <15%—were included. Primary analyses prespecified global OCT measures (average RNFLT and average GCIPLT) and global VF indices (MD and VFI). The 24−2 protocol was selected as the primary VF measure to enable uniform comparison across both disease groups; sectoral OCT–VF mapping and macula-enriched perimetry (Humphrey 10−2 and 24-2C protocols) were not uniformly available and therefore were not included in the primary analyses.

OCT images were obtained using Cirrus HD-OCT (Carl Zeiss Meditec). RNFLT and GCIPLT were measured using the optic disc cube and macular cube protocols, respectively. OCT images were evaluated for quality using OSCAR-IB [13] and APOSTEL 2.0 standards [14]. Peripapillary RNFL and macular GCIPLT were recorded at initial evaluation and during at least 6 months of follow-up. Scans were excluded if they demonstrated signal strength <6, decentration, segmentation artifacts, or motion artifacts. Scans showing microcystic inner nuclear layer changes, which can occur in ON and may confound RNFL and GCIPL measurements, were also excluded. Segmentation boundaries were manually corrected by a single masked examiner (H.K.). For ON eyes in the stable phase, post-edema RNFLT and GCIPLT measurements were obtained from scans showing clear resolution of acute inflammatory changes, with temporal separation of ≥6 months from acute onset combined with rigorous quality control ensuring analyzed measurements represent true atrophic changes.

### Statistical analysis

Statistical analyses were performed using SAS (version 9.4, SAS Institute, Cary, NC, USA) and R (version 4.3.1, R Core Team, International collaboration). Continuous variables are presented as means ± standard deviations and compared using independent *t* tests or Mann–Whitney U tests depending on normality. Categorical variables were compared using chi-square or Fisher’s exact tests.

Between-disease slope differences were tested in the full sample using continuous pooled models with a Group × Structure interaction (structure: RNFLT or GCIPLT), with coefficient inference based on heteroskedasticity-consistent (HC3) robust standard errors to accommodate plausible heteroskedasticity and unbalanced group sizes.

Within each disease group, simple linear regressions (ordinary least squares [OLS]) of VF indices (MD or VFI) on OCT parameters (average RNFLT or GCIPLT) were fit to quantify structure–function slopes.

Univariable analyses were performed to assess associations between OCT-derived structural parameters and visual function indices (MD and VFI) by OLS regression. A stepwise selection method was adopted with an entry p < 0.2 and a stay p < 0.05 to build a multivariable model. Age (years) was included as a covariate in multivariable models to account for potential age-related effects on structural and functional measures. To address age imbalance between groups, 1:2 PS matching (PSM) was performed, matching ON to POAG patients on age, sex, hypertension (HTN), and diabetes mellitus (DM) with a caliper of 0.20 × SD of the logit of the PS. Balance after matching was assessed using standardized mean differences (SMD), with SMD < 0.1 considered well-balanced. Interaction analyses were repeated in the PSM-matched cohort as the primary age-adjusted analysis. A pre-specified sensitivity analysis was performed restricting the sample to non-diabetic participants (ON: n = 45; POAG: n = 124) to evaluate whether differential diabetes prevalence drove the observed structure-function dissociation.

As secondary analyses, interval-wise comparisons between ON and POAG across RNFLT and GCIPLT ranges were assessed with Mann–Whitney U tests, and p-values were interpreted with caution given smaller ON counts in thicker ranges. Two-sided p < 0.05 was considered statistically significant.

## Results

### Baseline characteristics

A total of 194 patients were included: 47 with ON and 147 with POAG. Baseline characteristics are summarized in Table 1. Patients with POAG were significantly older (56.31 ± 15.15 vs. 37.63 ± 15.72 years, p < 0.001), had higher baseline IOP (IOP; 18.47 ± 5.00 vs. 15.55 ± 2.83 mmHg, p = 0.002), and were more likely to have DM (15.9% vs. 2.1%, p = 0.013) than patients with ON. Other parameters such as sex, laterality, axial length, and blood pressure were similar between the two groups. Among patients with ON, 70.2% exhibited contrast enhancement of the optic nerve on MRI. Serologic testing revealed ANA positivity in 21.3%, anti- AQP4 antibody positivity in 19.2%, and anti-MOG positivity in 10.6%. (S1 Table).

**Table 1:** Comparison of baseline characteristics.

Parameters	Optic neuritis (n = 47 eyes)	Glaucoma (n = 147 eyes)	p-value
Age (years)	37.63 ± 15.72	56.31 ± 15.15	<0.001†*
Sex (M/F) (patients)	14 (29.8%) / 33 (71.2%)	57 (38.8%) / 90 (62.2%)	0.266
Laterality (R/L) (eyes)	21 (44.7%) / 26 (56.3%)	78 (53.1%) / 69 (46.9%)	0.317
Follow-up duration (months)	47.56 ± 31.25	56.67 ± 39.36	0.239†
			
Axial length (mm)	24.39 ± 1.71	24.66 ± 2.02	0.685†
IOP, baseline (mmHg)	15.55 ± 2.83	18.47 ± 5.00	0.002†*
			
HTN (cases) (%)	6 (12.8%)	36 (24.8%)	0.082†
SBP (mmHg)	124.00 ± 12.91	126.24 ± 13.73	0.332
DBP (mmHg)	77.27 ± 12.22	74.44 ± 10.95	0.142
MAP (mmHg)	92.84 ± 11.48	91.71 ± 8.53	0.539
DM (cases) (%)	1 (2.1%)	23 (15.9%)	0.013†*
BMI (kg/m^2^)	23.3 ± 4.24	23.79 ± 3.64	0.323†

Continuous variables were analyzed using the independent *t* test and †Mann–Whitney U-test. Categorical variables are presented using descriptive statistics as numbers (%) and were compared using the χ^2^ test; *p < 0.05.

IOP, intraocular pressure; HTN, hypertension; SBP, systolic blood pressure; DBP, diastolic blood pressure; MAP, mean arterial pressure; DM, diabetes mellitus; BMI, body mass index.

### Treatment outcomes in patients with ON

The treatment outcomes for patients with ON were assessed over a follow-up period of at least 6 months. The results showed significant functional recovery. Mean BCVA improved from 0.81 ± 0.91 logMAR to 0.16 ± 0.46 logMAR (p < 0.001), and all VF indices showed significant improvement: MD improved from −15.35 ± 12.67 dB to −2.13 ± 2.58 dB (p < 0.001), VFI increased from 56.11% to 95.75% (p < 0.001), and PSD improved from 5.77 ± 4.14 dB to 2.83 ± 2.67 dB (p < 0.001).

Despite functional recovery, OCT revealed significant structural deterioration, with average RNFLT decreasing from 125.37 ± 54.84 µm to 76.05 ± 14.39 µm (p < 0.001) and average GCIPLT from 74.43 ± 14.21 µm to 68.80 ± 12.55 µm (p < 0.001) (S2 Table). On stable, post-edema scans, RNFLT and GCIPLT remained outside the device’s age-adjusted normative limits.

### Comparison of structure and function between ON and POAG groups

We compared patients with ON stabilized after treatment and those with POAG (Table 2). Average RNFLT and GCIPLT were not significantly different between the two groups (RNFLT: 76.20 ± 14.06 vs. 74.51 ± 12.88 µm, p = 0.450; GCIPLT: 68.07 ± 11.75 vs. 68.59 ± 9.60 µm, p = 0.852). However, patients with ON had significantly better VF performance: MD was −2.26 ± 2.65 dB in ON compared with −7.32 ± 6.88 dB in POAG (p < 0.001), VFI was 95.48% ± 6.96 in ON compared with 80.43% ± 20.81 in POAG (p < 0.001), and PSD was significantly lower in ON (p < 0.001). Quadrant analysis revealed characteristic inferior RNFL loss in patients with POAG, consistent with early glaucomatous damage. In contrast, patients with ON exhibited more temporally focused thinning. GCIPL reduction in the POAG group was more pronounced in the inferotemporal macular sectors, whereas the ON group showed more homogeneous thinning patterns.

**Table 2:** Comparison of VF indices and OCT measurements between the optic neuritis after remission and glaucoma groups.

Parameters	Optic neuritis after remission (n = 47 eyes)	Glaucoma (n = 147 eyes)	p-value
Visual field indices			
MD (dB)	−2.26 ± 2.65	−7.32 ± 6.88	<0.001†*
PSD (dB)	2.94 ± 2.81	6.71 ± 4.38	<0.001†*
VFI (%)	95.48 ± 6.96	80.43 ± 20.81	<0.001†*
OCT measurements			
RNFLT, average (μm)	76.20 ± 14.06	74.51 ± 12.88	0.450
Superior Temporal Inferior Nasal	89.41 ± 23.70	90.36 ± 21.93	0.802
	57.96 ± 16.86	62.60 ± 15.94	0.114†
	97.93 ± 24.78	79.43 ± 21.51	<0.001†*
	59.20 ± 10.73	65.62 ± 13.32	0.007†*
GCIPLT, average (μm)	68.07 ± 11.75	68.59 ± 9.60	0.852†
Superior	68.39 ± 12.69	72.14 ± 11.89	0.065†
Superotemporal	68.52 ± 10.94	69.33 ± 11.70	0.680
Inferotemporal	69.46 ± 11.05	62.05 ± 11.61	<0.001†*
Inferior	66.78 ± 11.49	63.59 ± 10.55	0.108†
Inferonasal	66.89 ± 13.90	69.41 ± 11.49	0.258†
Superonasal	68.87 ± 14.11	74.69 ± 12.48	0.014†*

MD, mean deviation; PSD, pattern standard deviation; VFI, visual field index; OCT, optical coherence tomography; RNFLT, retinal nerve fiber layer thickness; GCIPLT, ganglion cell inner plexiform layer thickness.

*p < 0.05; †Mann–Whitney U test.

### Structure and function correlation in the ON and glaucoma groups

**Fig 1:**
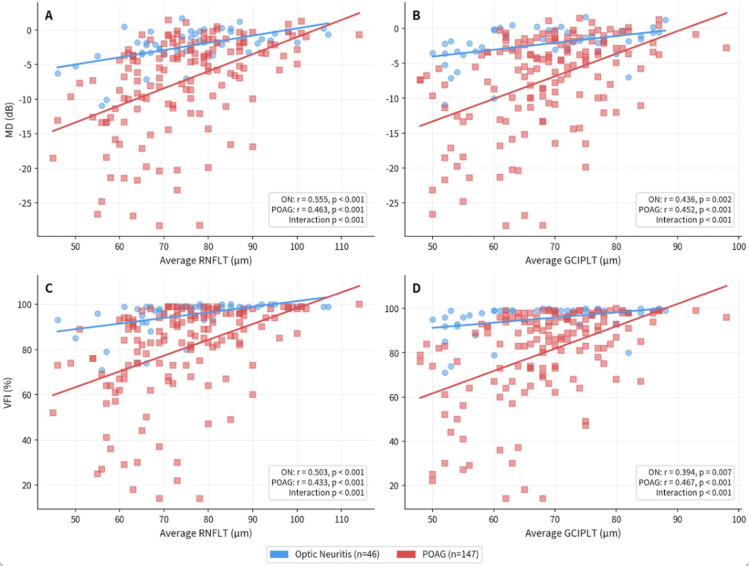
Comparison of the relationship among VF indices and OCT measurements in the ON and glaucoma groups using linear regression analyses. Linear regression analyses of MD and average RNFLT (A) and average GCIPLT (B) show significant differences between the ON and glaucoma groups. Similarly, the analysis of VFI with RNFLT (C) and GCIPLT (D) shows significant differences between the two groups. MD, mean deviation; VFI, visual field index; OCT, optical coherence tomography; RNFLT, retinal nerve fiber layer thickness; GCIPLT, ganglion cell-inner plexiform layer thickness; ON, optic neuritis; VF, visual field.

We conducted a series of correlation and linear regression analyses to assess differences in the relationship between VF indices and OCT measurements in the ON and POAG groups. Fig 1 illustrates the relationship among GCIPLT, RNFLT, and VF indices in the two groups. In the POAG group, VF indices decreased as the values of OCT measurements decreased. Specifically, lower GCIPLT and RNFLT were associated with worse MD and VFI.

Meanwhile, in the ON group, VF indices were maintained despite decreases in the values of OCT measurements. Additionally, the regression lines for the ON group exhibited a relatively stable relationship between the OCT measurements and VF indices. Within-group OLS regression confirmed significantly different slopes between the two groups across all four OCT-VF pairs (all P < 0.001): POAG showed steeper slopes than ON for both RNFLT (MD: β = 0.247 vs. 0.105; VFI: β = 0.700 vs. 0.249) and GCIPL (MD: β = 0.324 vs. 0.098; VFI: β = 1.012 vs. 0.233). Group-specific slopes and model R² are provided in Table 3, panel A.

**Table 3:** OLS regression analyses to identify factors associated with the difference in VF-OCT relationships.

A. Within-group OLS slopes								
Outcome	Optic neuritis (n=46 eyes)			Glaucoma (n=147 eyes)				
	β	95% CI	R^2^	β	95% CI	R^2^		
MD ~ RNFLT	0.105	0.057–0.152	0.308	0.247	0.170–0.325	0.214		
MD ~ GCIPLT	0.098	0.037–0.160	0.190	0.324	0.219–0.429	0.204		
VFI ~ RNFLT	0.249	0.119–0.379	0.253	0.700	0.461–0.939	0.188		
VFI ~ GCIPLT	0.233	0.068–0.399	0.155	1.012	0.697–1.326	0.218		
**B. Factors associated with VF-OCT relationships (OLS regression)**								
**Variable**	**Optic neuritis**				**Glaucoma**			
**Analysis of MD**	**Univariate** **analysis**	**Multivariate analysis 1**	**Multivariate** **analysis 2**	**VIF**	**Univariate** **analysis**	**Multivariate analysis 1**	**Multivariate analysis 2**	**VIF**
Age	0.301	0.721	-	1.62	0.004*	0.002*	0.001*	1.17
HTN	0.049*	0.294	-	1.81	0.231	0.518	-	1.27
DM	0.700	0.559	-	1.23	0.838	0.305	-	1.29
RNFLT, average	<0.001*	0.052	< 0.001*	2.97	<0.001*	0.008*	0.012*	1.93
GCIPLT, average	0.002*	0.656	-	2.97	<0.001*	0.005*	0.002*	1.96
**Analysis of VFI**	**Univariate analysis**	**Multivariate analysis 1**	**Multivariate analysis 2**	**VIF**	**Univariate** **analysis**	**Multivariate analysis 1**	**Multivariate analysis 2**	**VIF**
Age	0.215	0.310	-	1.62	0.001*	< 0.001	< 0.001*	1.17
HTN	0.603	0.404	-	1.81	0.145	0.388	-	1.27
DM	0.685	0.647	-	1.23	0.777	0.251	-	1.29
RNFLT, average	0.093	0.039*	0.093	2.97	<0.001*	0.057	-	1.93
GCIPLT, average	0.701	0.188	-	2.97	<0.001*	0.001*	< 0.001*	1.96

OLS, Ordinary least squares; CI, confidence interval; MD, mean deviation; VFI, visual field index; HTN, hypertension; DM, diabetes mellitus; RNFLT, retinal nerve fiber layer thickness; GCIPLT, ganglion cell inner plexiform layer thickness; VIF, variance inflation factor.

Multivariable analysis 1 included all five variables, and multivariable analysis 2 used a stepwise selection method with an entry p < 0.2 and a stay p < 0.05 to identify the most significant variables.

*p < 0.05.

Interval-wise comparisons across RNFLT and GCIPLT ranges are presented in S2 and S3 Figs. No significant differences were observed in MD and VFI between the ON and POAG groups (p = 0.272) for a GCIPLT ≥80 μm. However, significant differences were observed between the two groups (all p < 0.05) for other ranges. Similarly, no significant differences were observed in MD between the ON and POAG groups (p = 0.177) for an RNFLT >90 μm. However, significant differences were found in MD for other RNFLT ranges and in VFI across all RNFLT groups between the two conditions (all p < 0.05).

**Fig 2:**
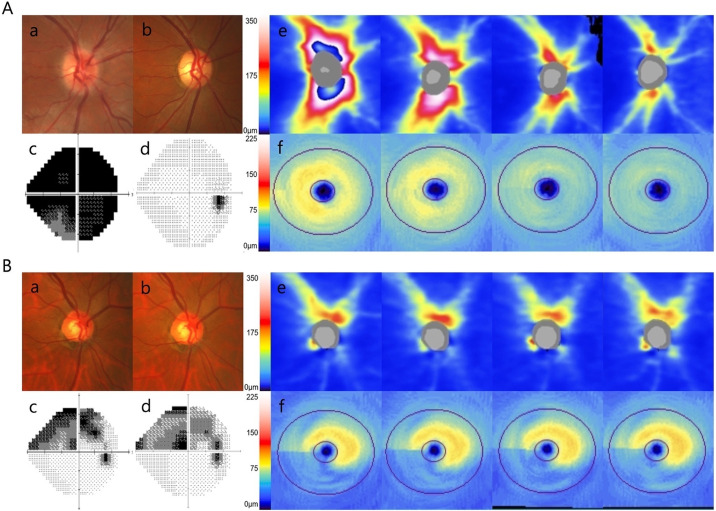
Representative cases of ON and glaucoma patients. A representative patient with ON in the right eye (A). FP images of the optic disc at the initial visit (a) and 6 months post-treatment (b). The VF pattern deviation map was fully recovered after 6 months of treatment (d) compared with that at the initial visit (c). However, following ON, RNFL thickness (e) and GCIPL thickness (f) significantly decreased. A representative patient with glaucoma in the right eye (B). During the 6-month follow-up, FP (a, b) and the RNFL (e) and GCIPL maps (f) showed a similar level of glaucomatous change. The VF pattern deviation map (c, d) showed the corresponding VF defects. Notably, despite comparable post-treatment RNFL thinning (ON: 66 µm vs. POAG: 63 µm), the ON patient demonstrated markedly better visual field performance, illustrating the disease-specific structure–function dissociation described in this study. FP, fundus photography; RNFL, retinal nerve fiber layer; GCIPL, ganglion cell-inner plexiform layer; ON, optic neuritis; VF, visual field.

Factors associated with VF–OCT relationships in each group are presented in Table 3, Panel B. In the analysis of MD, average RNFLT was a significant predictor in the ON group (p < 0.001). In the POAG group, age (p = 0.001), average RNFLT (p = 0.012), and average GCIPLT (p = 0.002) were significant predictors. For VFI, age (p < 0.001) and average GCIPLT (p < 0.001) were significant in the POAG group; no significant predictors were identified in the ON group, although average RNFLT showed a trend toward significance (p = 0.093). Representative cases are shown in Fig. 2.

### Propensity score matching and sensitivity analyses of group differences in structure–function relationships

To account for potential confounding by differences in age, sex, HTN, and DM between the two groups, we performed 1:2 PSM (Table 4). Of the 46 ON eyes and 147 POAG eyes eligible for matching, 29 ON eyes and 58 POAG eyes were successfully matched. After matching, all four covariates were well balanced between groups, with absolute SMD (|SMD|) below 0.10 for each covariate (Age |SMD| = 0.014; Sex |SMD| = 0.035; HTN |SMD| = 0.043; DM |SMD| = 0.000), as shown in S4 Fig.

**Table 4:** Group × structure interaction analyses of structure–function relationships.

Variable	Model 1 (Crude)		Model 2 (Age-adjusted)	
	Δβ (95% CI)	p-value	Δβ (95% CI)	p-value
MD – RNFLT	0.143 (0.061, 0.224)	< 0.001*	0.140 (0.058, 0.223)	< 0.001*
MD – GCIPL	0.225 (0.105, 0.346)	< 0.001*	0.216 (0.095, 0.336)	< 0.001*
VFI – RNFLT	0.451 (0.209, 0.693)	< 0.001*	0.443 (0.203, 0.683)	< 0.001*
VFI – GCIPL	0.779 (0.406, 1.151)	< 0.001*	0.747 (0.383, 1.111)	< 0.001*
**Variable**	**Model 3 (1:2 PSM)**		**Model 4 (Non-DM subgroup)**	
	**Δβ (95% CI)**	**p-value**	**Δβ (95% CI)**	**p-value**
MD – RNFLT	0.130 (0.013, 0.246)	0.029*	0.138 (0.055, 0.222)	0.001*
MD – GCIPL	0.262 (0.073, 0.451)	0.007*	0.228 (0.097, 0.360)	< 0.001*
VFI – RNFLT	0.396 (0.056, 0.736)	0.023*	0.423 (0.175, 0.670)	< 0.001*
VFI – GCIPL	0.824 (0.250, 1.397)	0.005*	0.777 (0.368, 1.185)	< 0.001*

Model 1: unadjusted (ON n = 46, POAG n = 147). Model 2: age-adjusted (ON n = 46, POAG n = 147). Model 3: 1:2 propensity score-matched cohort (ON n = 29, POAG n = 58). Model 4: non-diabetic subgroup (ON n = 45, POAG n = 124).

Δβ = difference in structure–function slope (POAG minus ON) derived from pooled OLS regression with group × structure interaction term; positive values indicate a steeper slope in the POAG group. HC3 robust standard errors.

*p < 0.05.

MD, mean deviation; VFI, visual field index; RNFLT, retinal nerve fiber layer thickness; GCIPL, ganglion cell-inner plexiform layer thickness; OLS, ordinary least squares; CI, confidence interval; PSM, propensity score matching; DM, diabetes mellitus; HC3, heteroskedasticity-consistent robust.

**Fig 3:**
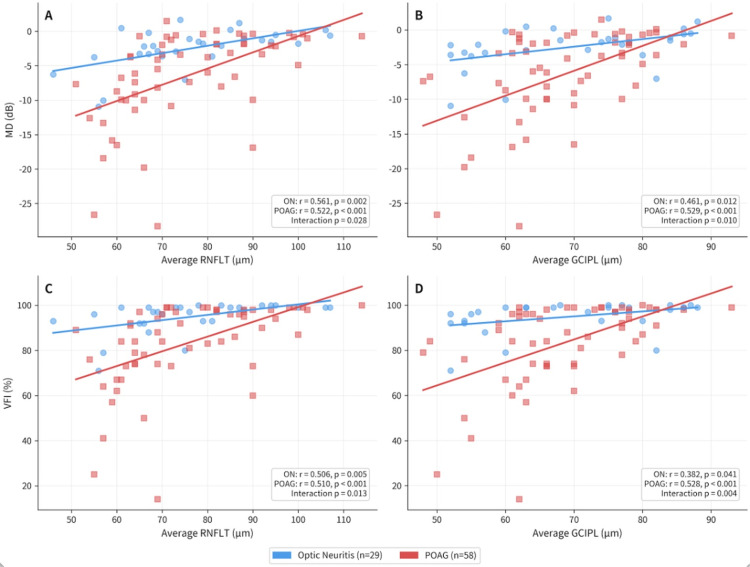
Structure–function relationships in the PSM cohort (ON n = 29, POAG n = 58). Linear regression analyses of MD with average RNFLT (A) and average GCIPL (B), and of VFI with average RNFLT (C) and average GCIPL (D), demonstrate significantly different slopes between the ON and POAG groups after 1:2 PSM. In all four panels, the POAG group showed a steeper structure–function slope than the ON group, and the group × structure interaction remained statistically significant (all interaction p < 0.05). PSM, propensity score matching; MD, mean deviation; VFI, visual field index; RNFLT, retinal nerve fiber layer thickness; GCIPLT, ganglion cell–inner plexiform layer thickness; ON, optic neuritis; POAG, primary open-angle glaucoma; PSM, propensity score matching.

**Fig 4:**
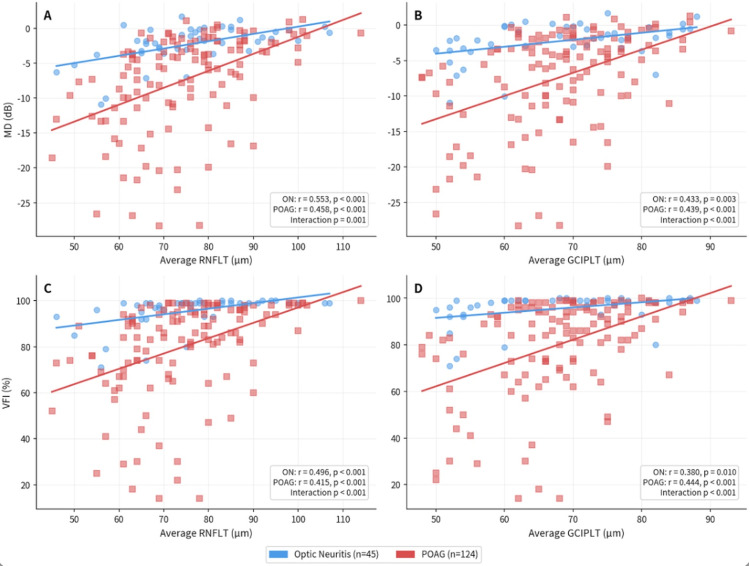
Structure–function relationships in the non-diabetic subgroup (ON n = 45, POAG n = 124). Linear regression analyses of MD with average RNFLT (A) and average GCIPL (B), and of VFI with average RNFLT (C) and average GCIPL (D), demonstrate consistently different slopes between the ON and POAG groups in patients without DM. In all four panels, the POAG group showed a steeper structure–function slope than the ON group, and the group × structure interaction remained statistically significant (all interaction p ≤ 0.001). MD, mean deviation; VFI, visual field index; RNFLT, retinal nerve fiber layer thickness; GCIPLT, ganglion cell–inner plexiform layer thickness; ON, optic neuritis; POAG, primary open-angle glaucoma; DM, diabetes mellitus.

Fig 3 illustrates the structure–function relationships in the matched cohort. The divergence between ON and POAG was preserved across all four OCT–VF pairs: in the POAG group, both RNFLT and GCIPL thickness remained significantly associated with MD and VFI, whereas the ON group continued to show a relatively flat slope, maintaining preserved VF indices despite substantial OCT thinning. This pattern was further confirmed in the non-diabetic subgroup (ON n = 45, POAG n = 124), in which the same divergence was consistently observed across all four variable pairs (Fig. 4), indicating that the observed structure–function dissociation is unlikely to be explained by the differential prevalence of diabetes mellitus between the two groups.

Formal interaction testing confirmed that the difference in slopes between the two groups remained statistically significant after matching. The POAG group showed a consistently steeper structure–function slope than the ON group for all four variable pairs: MD–RNFLT, MD–GCIPL, VFI–RNFLT, VFI–GCIPL. To further assess the robustness of these findings, we examined the interaction across three additional models (Table 4): an unadjusted full-cohort model (Model 1), an age-adjusted model (Model 2), and a non-diabetic subgroup analysis (Model 4). In all three, the group × structure interaction remained significant (all p < 0.001), and the magnitude of the slope difference was consistent with that observed in the matched cohort, collectively supporting the robustness of the group difference in structure–function relationships.

## Discussion

This study highlights a fundamental difference in the structure–function relationship between ON and POAG. Although OCT-derived measures were comparable in both groups after ON recovery, patients with ON retained significantly better VF performance. This structure–function dissociation emphasizes the disease-specific nature of retinal neurodegeneration and functional compensation.

The deliberate selection of non-progressive POAG patients was essential for fair comparison with post-inflammatory stable ON patients. Including progressive glaucoma would fundamentally alter the research question from structure-function relationships to progression patterns. Our findings demonstrate that GCIPLT of 70 μm may indicate severe functional impairment in stable glaucoma but relatively preserved function in post-ON eyes. This distinction is only meaningful when comparing stable states and provides evidence-based guidance for OCT interpretation in routine practice.

The observed age difference reflects authentic epidemiological characteristics rather than methodological bias. ON typically affects younger adults associated with demyelinating diseases, while POAG predominantly affects older adults. Importantly, disease-specific differences remained significant after age adjustment, demonstrating that structure-function dissociation is disease-intrinsic rather than age-related. To further address potential confounding by sex, HTN, and DM, we performed PSM, which yielded a balanced cohort in which the group difference in structure–function slopes remained statistically significant across all four OCT–VF pairs (Table 4, Model 3). The consistency of findings across unadjusted, age-adjusted, PS–matched, and non-diabetic subgroup analyses (Table 4, Models 1–4) collectively confirms that the dissociation is disease-intrinsic rather than driven by demographic or comorbidity-related confounding. This strengthens the validity of our conclusions and enhances practical utility for clinical decision-making.

In POAG, structural thinning closely correlates with visual dysfunction, which aligns with the progressive and irreversible RGC loss [15,16]. RNFLT and GCIPLT represent direct markers of axonal and soma loss in POAG, and both were strong independent predictors of VF parameters in our cohort, reinforcing the utility of OCT as a surrogate for functional impairment in glaucoma [17]. Additionally, chronic atrophy in glaucoma involves not only IOP-related mechanical damage but also vascular dysregulation, endothelial dysfunction, and glial cell impairment, particularly Müller cells and astrocytes, exacerbating neuroaxonal damage [18–20]. In contrast, after the acute phase of ON, inner retinal thinning can reflect a mixture of edema resolution and post-inflammatory remodeling; because conduction recovery, remyelination, and neuroplastic compensation may outpace structural normalization, VF can remain relatively preserved at comparable OCT thickness levels [20–26].

Topographically, glaucoma preferentially damages the superior and inferior arcuate bundles, whereas ON often involves papillomacular and macular pathways with macula-predominant GCIPL change; these distinct pathways likely yield different sectoral signatures on OCT and perimetry [15–17]. Accordingly, sectorally oriented OCT–VF interpretation and macula-enriched testing (Humphrey 10−2 or 24-2C) may better capture localized associations, particularly in ON where central macular involvement is common [11,12,27,28].

Several hypotheses explain the preserved function in ON despite anatomical thinning. Neuroplasticity, synaptic reorganization, and compensatory visual pathway activity have been suggested as contributing mechanisms [21]. Surviving RGCs and supporting Müller glia may maintain function even in the presence of reduced inner retinal thickness [22–24]. These concepts are supported by recent longitudinal OCT studies showing continued RNFL decline in eyes with improved or stable VF performance after ON [20]. In addition, GCIPL loss after ON can partly reflect neuronal soma or dendritic shrinkage rather than outright cell death, further contributing to a shallower structure–function slope compared with glaucoma [20–26]. This temporal decoupling therefore likely underlies the between-disease differences we observed.

This dissociation was especially apparent in the lower structural ranges. Patients with ON demonstrated better VF indices than those with POAG even at RNFLT <90 µm and GCIPLT <80 µm—ranges typically associated with moderate-to-severe functional loss in glaucoma. Conversely, in glaucoma, comparable macular GCIPL thinning more often corresponds to irreversible ganglion-cell loss with chronic neurovascular and glial dysfunction; arcuate bundle damage also aligns closely with the 24–2 sampling grid, which can make structure–function coupling appear steeper [11,12,15–20,28]. Consistent with this vascular contribution, OCT-angiography studies have shown that reduced peripapillary and macular vessel density is associated with functional loss in glaucoma [20,29]. Differences in dynamic range and measurement floor for OCT parameters should also be considered when interpreting slopes, as these factors can accentuate coupling in glaucoma relative to ON [31,32].

Clinically, OCT parameters must be interpreted within the context of the underlying disease. A GCIPLT of 70 µm may indicate advanced glaucomatous damage but could represent post-inflammatory remodeling in ON with preserved function. As OCT continues to be widely adopted in general practice, this distinction is essential to prevent misclassification and overtreatment. Rather than relying on a single thickness threshold, diagnosis-aware interpretation should guide OCT–VF assessment across optic neuropathies; as noted above, sectoral OCT–VF correspondence and macula-enriched perimetry can better capture localized associations, and vascular adjuncts (e.g., OCT-A vessel-density metrics) may add value when global averages are similar [11,12,27–30]. These practical steps are also consistent with ongoing efforts to personalize OCT interpretation (e.g., individualized RNFL norms, texture/feature-based OCT, and composite structure–function indices) and may reduce misclassification in eyes with overlapping OCT ranges [33–35].

This study has several limitations. First, we acknowledge the higher prevalence of DM in our POAG cohort. Recent evidence suggests that DM can induce subclinical retinal neurodegeneration, potentially representing a minor unmeasured confounder in structural measurements [36]. However, the group × structure interaction remained significant in the non-diabetic subgroup analysis (Table 4, Model 4), indicating that DM alone cannot account for the observed structural–functional dissociation. Second, the retrospective design restricted inclusion of complementary functional tests such as contrast sensitivity and color vision, which may provide additional insights into ON recovery. Third, the ON group was relatively small and heterogeneous, with varying inflammatory burden and serologic status. Fourth, central-field sampling was limited by routine 24−2 perimetry, and sectoral OCT-VF mapping was not uniformly available, potentially attenuating localized structure-function correlations. Fifth, microcystic inner nuclear layer changes were not systematically graded in this cohort. Finally, the intentional inclusion of only non-progressive POAG patients introduces a selection bias, as this cohort may not represent the broader glaucoma population. While this design decision was necessary to enable a stable, cross-sectional comparison with post-inflammatory ON eyes in remission and to isolate the structure–function relationship from the confounding effects of ongoing neurodegeneration, our findings may not generalize to patients with progressive or advanced glaucoma, in whom active axonal loss may further modify the structure–function slope. Future prospective studies incorporating stratified disease subtypes and advanced imaging techniques are warranted.

## Conclusions

Although both ON and POAG result in inner retinal thinning, the clinical interpretation of these structural changes must be disease-specific. Our findings demonstrate that VF performance cannot be reliably inferred from OCT alone in ON and that functional preservation may persist despite significant structural loss. These insights may refine diagnostic algorithms and support the development of condition-specific OCT thresholds or artificial intelligence-based interpretation systems for optic neuropathies.

## Supporting information

S1 Fig STROBE Flowchart of patient selection. (DOCX)

S2 Fig Comparisons of the relationship between VF Indices and OCT measurements across different GCIPLT intervals. (DOCX)

S3 Fig Comparisons of the relationship between VF Indices and OCT measurements across different RNFLT intervals. (DOCX)

S4 Fig Propensity score matching diagnostics. (DOCX)

S1 Table Initial clinical evaluation of optic neuritis. (DOCX)

S2 Table Treatment outcomes in patients with ON. (DOCX)

S1 File Underlying de-identified dataset used for the analyses in this study. (XLSX)
