# Supplementary figures and images for: Structure function relationships differ between optic neuritis and glaucoma with comparable optical coherence tomography findings
Source: PLoS One. 2026 Jul 16;21(7):e0353553. doi: 10.1371/journal.pone.0353553 (PMC13374924; doi:10.1371/journal.pone.0353553)

**Supporting Fig. 1 STROBE Flowchart of patient selection**

**
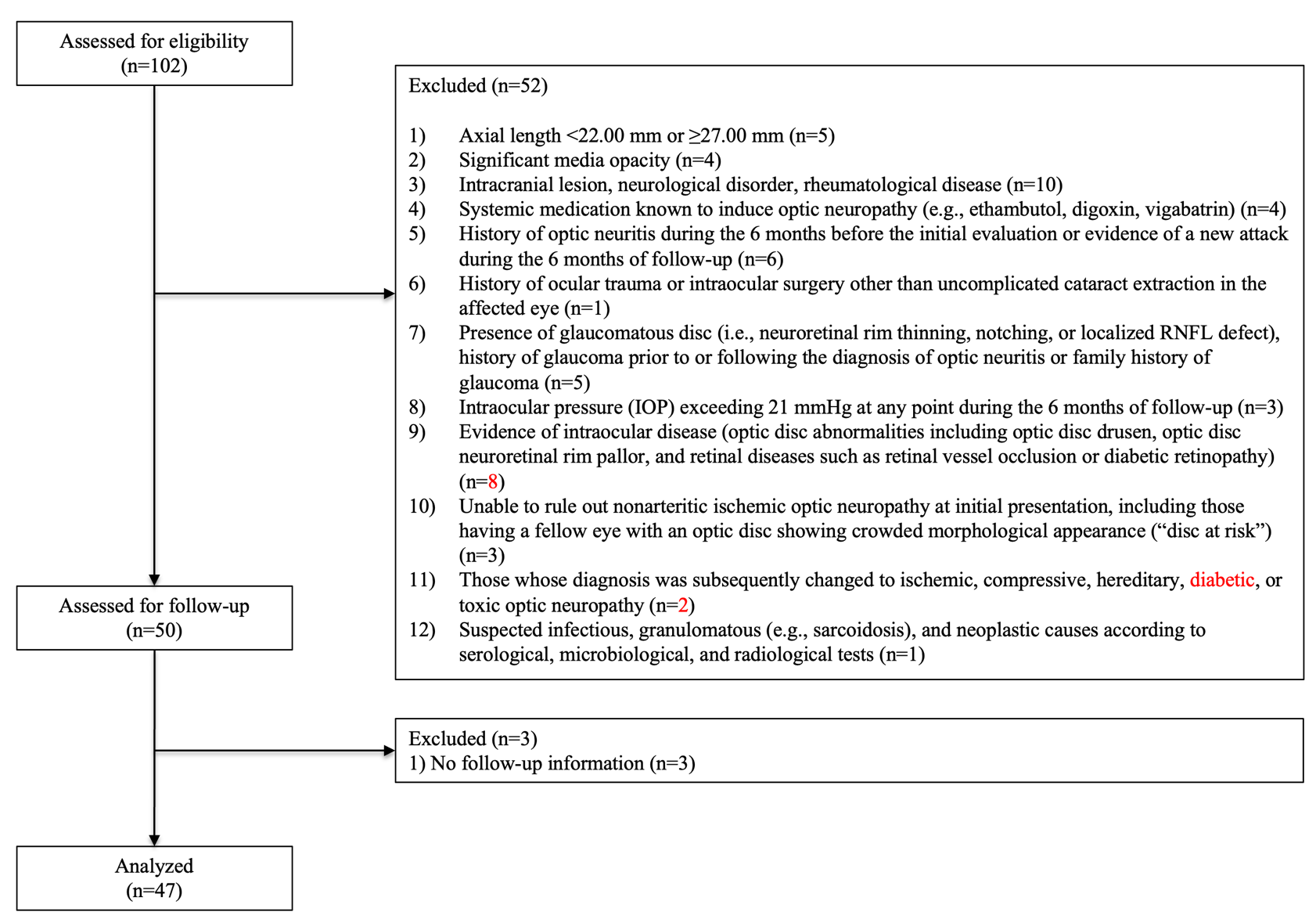
**

RNFL, retinal nerve fiber layer

Supplement: S1 Fig — (DOCX) [file pone.0353553.s001.docx]
