# Supplementary material for: Structure function relationships differ between optic neuritis and glaucoma with comparable optical coherence tomography findings
Source: PLoS One. 2026 Jul 16;21(7):e0353553. doi: 10.1371/journal.pone.0353553 (PMC13374924; doi:10.1371/journal.pone.0353553)
Supplement: S3 Fig — (DOCX) [file pone.0353553.s003.docx]

**Supporting Fig. 3 Comparisons of the relationship between VF Indices and OCT measurements across different RNFLT intervals**


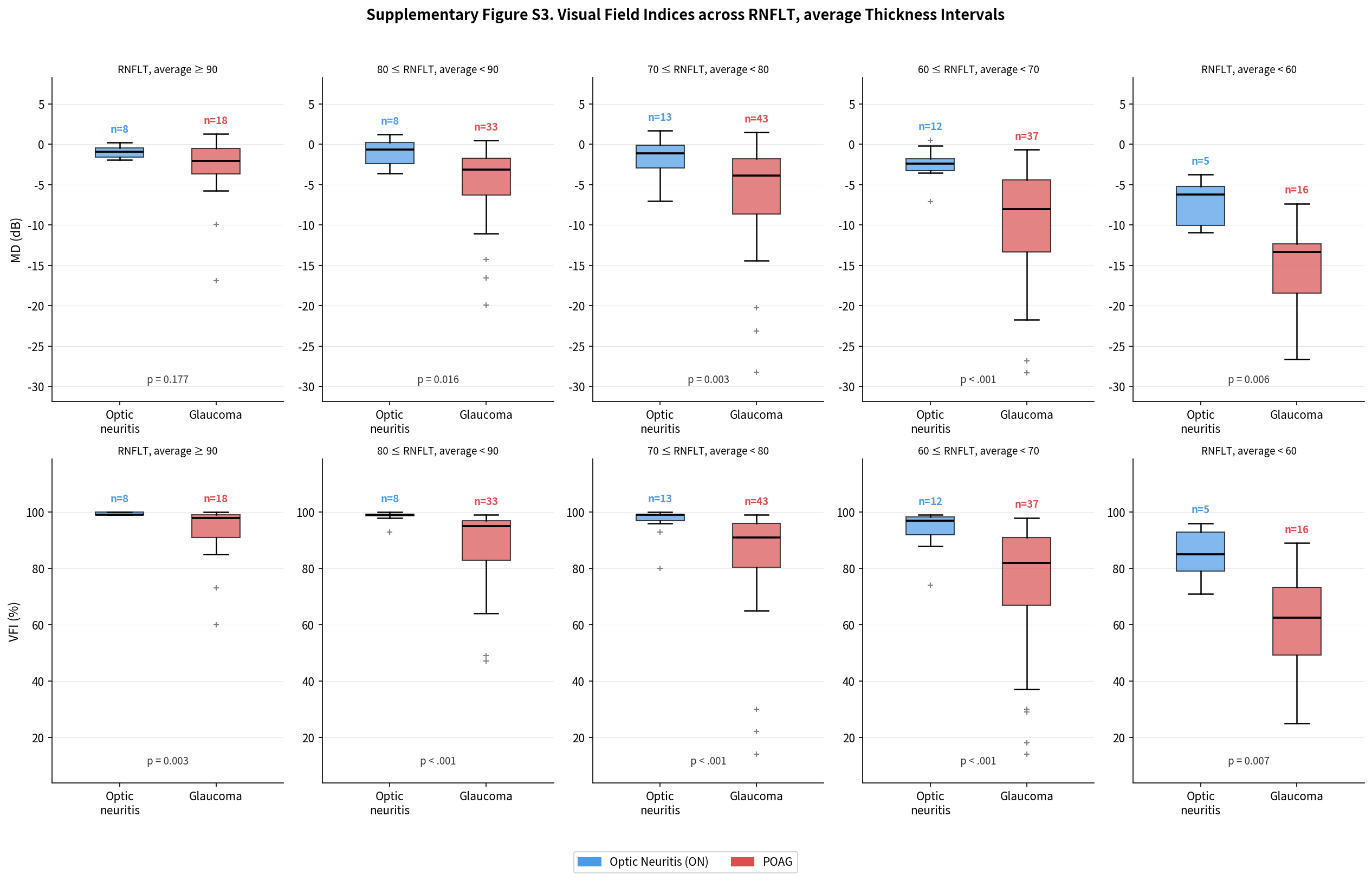


OCT, optical coherence tomography; RNFLT, retinal nerve fiber layer thickness; VF, visual field; VFI, visual field index; MD, mean deviation
