# Supplementary material for: Structure function relationships differ between optic neuritis and glaucoma with comparable optical coherence tomography findings
Source: PLoS One. 2026 Jul 16;21(7):e0353553. doi: 10.1371/journal.pone.0353553 (PMC13374924; doi:10.1371/journal.pone.0353553)
Supplement: S4 Fig — (DOCX) [file pone.0353553.s004.docx]

**Supporting Fig. 4 Propensity score matching diagnostics**

**
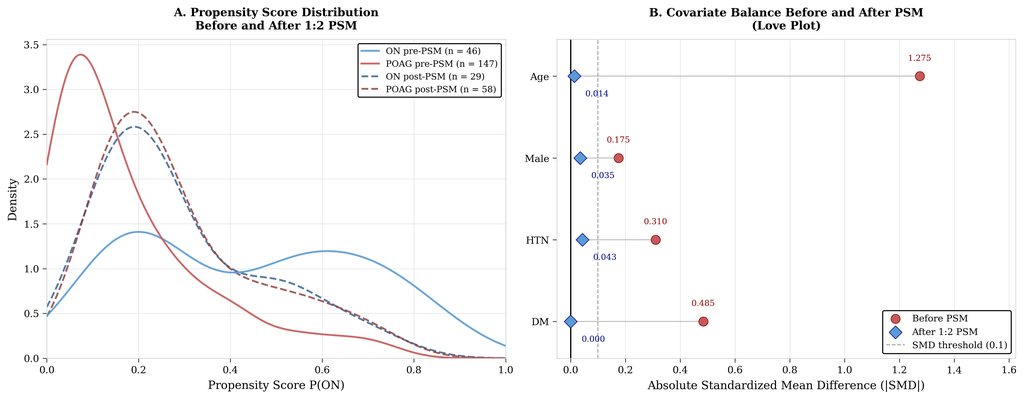
**

(A) Density plots of propensity scores before and after 1:2 nearest-neighbor propensity score matching for the optic neuritis (ON) and primary open-angle glaucoma (POAG) groups. Propensity scores were estimated using logistic regression with age, sex, hypertension, and diabetes mellitus as covariates. After matching, the distributions of propensity scores were substantially overlapping between groups. (B) Love plot showing absolute standardized mean differences (SMDs) for each covariate before and after matching. All covariates achieved |SMD| < 0.10 after matching (Age 0.014, Sex 0.035, hypertension 0.043, diabetes mellitus 0.000), indicating adequate covariate balance.

PSM, propensity score matching; ON, optic neuritis; POAG, primary open angle glaucoma
