# Supplementary material for: Structure function relationships differ between optic neuritis and glaucoma with comparable optical coherence tomography findings
Source: PLoS One. 2026 Jul 16;21(7):e0353553. doi: 10.1371/journal.pone.0353553 (PMC13374924; doi:10.1371/journal.pone.0353553)
Supplement: S1 Table — (DOCX) [file pone.0353553.s005.docx]

**Supporting Table 1. Initial clinical evaluation of optic neuritis**

| **Parameters** | **Optic neuritis**  **(n=47 eyes)** |
| --- | --- |
| RAPD (cases) (%) | 39 (84.78%) |
| Moving pain (cases) (%) | 23 (56.10%) |
| Color sense test (number of errors) | 9.9 ± 10.77 |
| MRI enhancement (cases) (%) | 33 (70.21%) |
| Serological evaluation |  |
| CRP (mg/L) | 2.09 ± 3.13 |
| ESR (mm/h) | 19.48 ± 20.55 |
| ANA (cases) (%) | 10 (21.28%) |
| Anti-MOG (cases) (%) | 5 (10.64%) |
| Anti-AQP4 (cases) (%) | 9 (19.15%) |
| CSF evaluation |  |
| CSF pressure (mmHg) | 166 ± 50.26 |
| CSF oligoclonal band (cases) (%) | 9 (19.15%) |

RAPD, relative afferent pupillary defect; MRI, magnetic resonance imaging; CRP, C-reactive protein; MOG, myelin oligodendrocyte glycoprotein; ESR, erythrocyte sedimentation rate; AQP4, aquaporin-4; ANA, anti-nuclear antibody; CSF, cerebrospinal fluid
