# Supplementary material for: Structure function relationships differ between optic neuritis and glaucoma with comparable optical coherence tomography findings
Source: PLoS One. 2026 Jul 16;21(7):e0353553. doi: 10.1371/journal.pone.0353553 (PMC13374924; doi:10.1371/journal.pone.0353553)
Supplement: S2 Table — (DOCX) [file pone.0353553.s006.docx]

**Supporting Table 2. Treatment outcomes in patients with ON**

| **Outcomes** | **Initial** | **Final** | **Difference** | **p-value** |
| --- | --- | --- | --- | --- |
| BCVA (logMAR) | 0.81 ± 0.91 | 0.16 ± 0.46 | -0.64 ± 0.90 | < 0.001^‡^* |
| IOP | 16.19 ± 4.70 | 14.21 ± 2.82 | -1.98 ± 4.37 | < 0.001^‡^* |
| Visual field indices |  |  |  |  |
| MD (dB) | -15.35 ± 12.67 | -2.13 ± 2.58 | 13.22 ± 11.69 | < 0.001^‡^* |
| PSD (dB) | 5.77 ± 4.14 | 2.83 ± 2.67 | -2.94 ± 4.54 | < 0.001* |
| VFI (%) | 56.11 ± 39.25 | 95.75 ± 6.61 | 39.64 ± 37.43 | < 0.001^‡^* |
| OCT measurements |  |  |  |  |
| RNFLT, average (μm) | 125.37 ± 54.84 | 76.05 ± 14.39 | -49.32 ± 54.75 | < 0.001^‡^* |
| Superior  Temporal  Inferior  Nasal | 162.54 ± 74.67 | 88.80 ± 23.99 | -73.73 ± 80.33 | < 0.001^‡^* |
|  | 87.22 ± 47.04 | 58.71 ± 16.65 | -28.51 ± 43.42 | < 0.001^‡^* |
|  | 151.51 ± 63.57 | 97.12 ± 25.29 | -54.39 ± 62.30 | < 0.001^‡^* |
|  | 98.46 ± 55.96 | 59.20 ± 11.09 | -39.27 ± 56.23 | < 0.001^‡^* |
| GCIPLT, average (μm) | 74.43 ± 14.21 | 68.80 ± 12.55 | -5.63 ± 11.80 | 0.001^‡^* |
| Superior | 75.83 ± 15.38 | 69.33 ± 13.64 | -6.50 ± 13.34 | 0.002^‡^* |
| Superotemporal | 75.90 ± 12.49 | 69.07 ± 12.10 | -6.83 ± 11.96 | 0.001^‡^* |
| Inferotemporal | 74.17 ± 14.86 | 69.50 ± 11.73 | -4.67 ± 11.12 | 0.003^‡^* |
| Inferior | 71.40 ± 16.77 | 67.27 ± 11.77 | -4.13 ± 13.37 | 0.017^‡^* |
| Inferonasal | 75.07 ± 15.15 | 68.17 ± 13.91 | -6.90 ± 13.36 | < 0.001^‡^* |
| Superonasal | 76.23 ± 16.19 | 70.30 ± 14.38 | -5.93 ± 13.07 | 0.002^‡^* |

ON, optic neuritis; BCVA, best corrected visual acuity; logMAR, logarithm of the minimum angle of resolution; IOP, intraocular pressure; MD, mean difference; PSD, pattern standard deviation; VFI, visual field index; OCT, optical coherence tomography; RNFLT, retinal nerve fiber layer thickness; GCIPLT, ganglion cell inner plexiform layer thickness. *p < 0.05; ^‡^Wilcoxon signed-rank test
